# Accessible and cost-effective deployment of environmental DNA (eDNA) samplers for sediment conducive to supporting community-based surveys

**DOI:** 10.1371/journal.pone.0342851

**Published:** 2026-02-24

**Authors:** Anna H. Dema, Ellika M. Crichton, Neha Acharya-Patel, Lauren C. Bergman, Michael J. Allison, Matthew T. Bonderud, Jacob J. Imbery, Clifford L.K. Robinson, Jacqueline R. Huard, Caren C. Helbing

**Affiliations:** 1 Department of Biochemistry and Microbiology, University of Victoria, Finnerty Road, Victoria, British Columbia, Canada; 2 Pacific Biological Station, Fisheries and Oceans Canada, Nanaimo, British Columbia, Canada; 3 Comox Valley Project Watershed Society, Rosewall Crescent, Courtenay, British Columbia, Canada; UTRGV: The University of Texas Rio Grande Valley, UNITED STATES OF AMERICA

## Abstract

The presence of at-risk, invasive, and sentinel species are measures of biodiversity, however it is often challenging to quickly gather reliable data through conventional, time-constrained surveying techniques. Environmental DNA (eDNA) detection is one method that has proven to be extremely useful for biomonitoring, particularly due to its non-invasive nature, cost efficiency, sensitivity, accuracy, and relative ease to carry out in the field. Conventional sediment sampling presents a challenge to obtain suitable representative samples and there is a need for easily accessible methods that are compatible with community-based monitoring activities and budgets. Herein, we introduce a “FloppE-Dip” method, a passive sampling approach that is compatible with larger sand sediment sample volumes up to 180 mL and utilizes easily accessible materials. We compared the FloppE-Dip method to conventional filtration through the detection of several species’ DNA from environmental samples using real-time quantitative PCR (qPCR). In a laboratory study using an American bullfrog (*Rana [Lithobates] catesbeiana*) tissue slurry, we determined optimal protocol parameters that were then applied in a field survey to identify beaches in coastal British Columbia used by the important forage fish, the Pacific sand lance (*Ammodytes personatus*). Of the 20 sampling sites, both FloppE-Dip and conventional filtration methods detected Pacific sand lance eDNA at 14 of them, albeit FloppE-Dip copies/L estimates were typically 5–10 times lower. Two sites returned no detections for both methods, and four sites returned low detections using conventional filtration methods where the FloppE-Dip method showed no detection. This discrepancy at low copy numbers may be rectified through increasing the number of samples taken per site and/or increasing the number of technical replicates. Overall, the FloppE-Dip method was more reliable than visual observation and is considerably faster and cheaper than filtration making it well-suited for general detection purposes. While other sediment types remain to be tested, the simplicity, efficiency, and use of readily available materials make FloppE-Dip a viable alternative for community-based monitoring programs, particularly given the time and budget constraints these programs often face.

## Introduction

The biomonitoring of ecosystems through close surveillance of organisms occupying different ecological niches is essential for assessing species that are at-risk, pathogenic, invasive, sentinel, or keystone. This in turn provides insights on the impacts of climate change and anthropogenic stressors. In recent years, environmental DNA (eDNA) detection has become a cornerstone approach in inferring species presence and occasionally, abundance [1–6]. eDNA refers to the genetic material that is released by organisms through several sources (e.g., fur/hair, saliva, skin cells, fecal matter) that is found in an environmental sample such as water, soil, sand, sediment, or air [7–9]. Conventional surveying methods are limited in detecting species with low population densities, inconspicuous species (i.e., nocturnal, cryptic, or well-concealed), and species that occupy difficult to survey habitats [10–14]. Moreover, some conventional methods involve capture of the animal which is highly invasive [15,16]. Challenges can also arise when extensive training or expertise is required, such as morphological identification [17,18], or if specialized or expensive equipment is required. eDNA offers an approach that can circumvent these limitations due to its non-disruptive nature to the species of interest and their habitat, its cost and time efficiency, its ability to detect organisms that cannot be directly observed at the time of surveying, its high sensitivity and accuracy, and the relative ease of sampling in the field [6,7,19,20]. Several studies have demonstrated successful integration of eDNA methods with community based or citizen scientist programs [21–24], however these examples are limited to the collection of water samples through filtration and still required expensive preservation methods or use of dangerous goods (e.g., freezing samples, preserving in ethanol).

Sampling from soil or sediment presents a particular challenge as these samples often contain inhibitors for detection [25], have heterogenous eDNA distribution, and require special handling, costly storage, and transport prior to DNA extraction [26]. While improved methods for collecting such eDNA samples exist [12,27–29], these approaches also typically require specialized equipment, immediate freezing, vacuum filtration, and costly sample freezing, storage, and transport before processing. These can present cost and logistical barriers for volunteer-led initiatives operating on limited budgets and in remote field locations.

Passive eDNA sampling offers an alternative approach to active filtration by allowing for the collection of eDNA at reduced effort and cost [2,30–33] and can therefore be adopted by community based monitoring or citizen scientist initiatives (e.g., Indigenous peoples, local communities, and general public) [18]. There is considerable interest in using passive sampling methods as an alternative to active filtration methods to reduce sampling cost and burden, particularly for citizen science initiatives [2,30–33]. While a wide range of different natural and artificial sampling media have been used including sponges, activated carbon, gauze, and nylon, glass, or cellulose ester filters [30–32,34], many are custom-made, not easily available, or suffer from logistical issues in preserving, handling, and extracting captured DNA (e.g., large clumps of wet gauze).

Herein, we test an accessible, cost-effective, and passive eDNA sampling technique which we named the “FloppE-Dip” method by directly comparing it to a conventional method for handling larger sediment volumes (150–180 mL) involving filtration. The FloppE-Dip method consists of agitating a small piece of positively charged nylon membrane in mixture of sediment and water, allowing the DNA to bind to the membrane for a brief amount of time, followed by preservation of the membrane in non-toxic silica beads [35]. We compared estimated DNA copy numbers of several target species in a series of paired design experiments: We first tested FloppE-Dip’s ability to bind and retain DNA by shaking the membrane in sediment samples spiked with American bullfrog (*Rana [Lithobates] catesbeiana*) tissue slurry and determined an optimal contact time. We then assessed FloppE-Dip’s performance in the field using intertidal sand sediments collected from 20 beaches used by the small forage fish, Pacific sand lance (*Ammodytes personatus*), an important indicator species known to spawn on intertidal habitats along the Salish Sea, British Columbia [13,36]. The present work demonstrates the potential of the FloppE-Dip method to facilitate community-based collection of samples, accommodate limited budgets, and enhance usability for scalable eDNA monitoring.

## Materials and methods

### FloppE-Dip protocol overview

**Fig 1:**
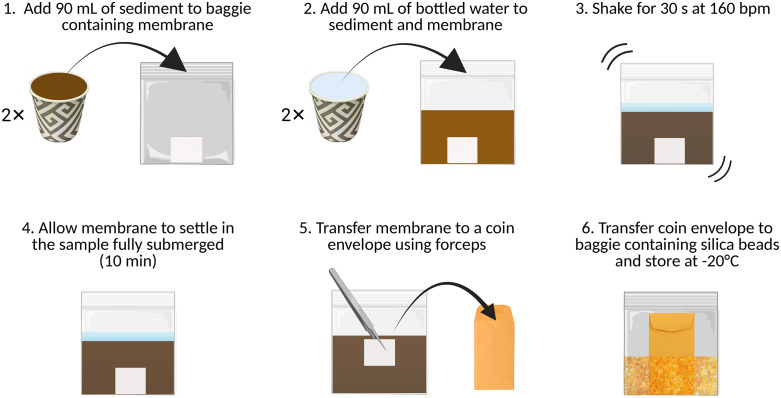
An overview of the FloppE-Dip protocol workflow. Resealable baggies each containing a single membrane are prepared in advance of field work in a laminar flow hood to avoid contamination. Each sample is collected with fresh materials (refer to Figure 4). As Sample collection is done with fresh cups and bottled water with clean hands or wearing nitrile gloves. Take particular care not to touch the membrane or inside of the cup or baggie with bare or contaminated hands at any point. In the field, 180 mL of sediment and 180 mL fresh bottled water (using a 90 mL paper cup twice for each) are added to a resealable baggie containing a 3 x 3 cm^2^ piece of membrane, the baggie shaken for 30 s at 160 bpm, allowed to settle for 10 min, and the membrane transferred to a manilla coin envelope using clean forceps. Users are encouraged to wear nitrile gloves when transferring the membrane to the coin envelope. The envelope is then put into a small resealable baggie containing silica beads and stored in a manual defrost freezer (-20°C) until the DNA is extracted.

A pictorial overview of the protocol is presented in Fig 1. The protocol described in this peer-reviewed article is published on protocols.io (10.17504/protocols.io.261ge8q5og47/v1) and is included for printing purposes as S1 File. As with any eDNA sampling protocol, best practices to reduce contamination should be followed [13] and users are strongly encouraged to refer to the minimum reporting requirements of eDNA surveys for information regarding survey design and reporting [37] and that the eDNA assay for intended use meets the minimum performance criteria [1]. For each sample to be collected, we compiled a basic sampling kit comprised of a 3 x 3 cm piece of membrane placed in a labelled re-sealable plastic baggie (small size freezer bag), a fresh 3-ounce (90 mL) paper cup, two forceps, ~ 400 mL fresh bottled or distilled (dH_2_O) water, and bleach (e.g., Clorox wipes, 10% (v/v) bleach) for decontaminating the forceps between samples. The present study used a high-strength nylon membrane positively charged with quaternary amine groups: Zeta-Probe membranes (Bio-Rad Laboratories, Mississauga, ON, Canada). Using disposable 90 mL paper cups, two level scoops of sediment (180 mL), and two full cups of fresh bottled water (180 mL) were added into the plastic baggie containing a membrane. The air was carefully removed and the baggie sealed. The baggie containing the sediment, water, and membrane was then shaken for 30 s at 160 beats per minute (bpm) (approximately 60–80 shakes) (Fig 1). To help keep the beat, practitioners were instructed to listen to a curated playlist of songs with a bpm of 160 (Table 1) and to verify shaking rate accuracy, a smartphone metronome application was utilized.

**Table 1:** A curated Spotify Playlist with songs at or near 160 bpm, the required shaking rate for FloppE-Dip samples (https://open.spotify.com/playlist/4L8gu97y0w6dzvLa3hRcLn?si=92c791f833ca4714).

Artist	Song	BPM
OutKast	Hey Ya!	159
The Beach Boys	Surfin’ USA	159
The Romantics	What I Like About You	160
John Travolta	Greased Lightnin’ – From “Grease”	159
Train	Drops of Jupiter (Tell Me)	159
The Neighbourhood	Stargazing	160
Blondie	One Way or Another	162

The membrane was allowed to settle in the sediment and water mixture for 10 min, ensuring it remained submerged. Clean forceps were used to retrieve the FloppE-Dip membrane from the baggie, transferred unfolded to a pre-labelled manila coin envelope (labelled with site ID, station ID, and date), and stored with silica beads at -20^o^C in a manual defrost freezer according to the protocol outlined previously [35]. Between samples, the forceps were cleaned with bleach, rinsed thoroughly with fresh bottled water, and air dried.

### Spiked sandy sediment experiment

#### Animal care and handling.

A single *R. catesbeiana* tadpole was used as a tissue source to create a standard slurry. The tadpole was euthanized using 0.1% (w/v) tricaine methanesulfonate (Syndel Laboratories, Nanaimo, BC, Canada) buffered in 25 nM sodium bicarbonate (Sigma-Aldrich, Oakville, ON. Canada).

#### Standard frog slurry creation.

All laboratory work was conducted in an amplicon free area, bench tops and equipment were cleaned with 10% (v/v) bleach followed by 70% (v/v) ethanol as necessary, and all molecular work was conducted in a laminar flow hood. A standard frog slurry was created using five previously frozen 4 mm biopsy punches from *R. catesbeiana* tadpole tailfins that had been preserved in RNAlater (ThermoFisher Scientific, Ottawa, ON, Canada). The biopsy punches were dried on KimWipes (ThermoFisher Scientific, Ottawa, ON, Canada) to remove any excess RNAlater solution and 140 mg tissue was placed into a 15 mL conical tube. Distilled water was then added to a total volume of 5 mL. Ten µL 50% Tween 20 detergent (Millipore Sigma Ltd., Oakville, ON, Canada) were added and the tissue was homogenized using a Heidolph DIAX 600 homogenizer (Wood Dale, IL). A drop of the slurry was visually inspected under a Nikon TMS contrast microscope (ThermoFisher Scientific, Ottawa, ON, Canada) to confirm complete cell lysis.

#### Field sampling protocol.

Sampling was conducted in the mid-intertidal area at Willows Beach (latitude: 48.430304, longitude: −123.304255) in Victoria, British Columbia on July 18, 2022. Quadruplicate 6-ounce (180 mL) sandy sediment samples were collected from five stations in resealable baggies and immediately placed in a cooler with ice. The samples were transported to the laboratory within two hours of sample collection. In the lab, each sediment sample was spiked by pipetting 200 L of the *R. catesbeiana* standard slurry directly into the sediment.

For the FloppE-Dip method, bottled water was added to the spiked sediment sample as described in the FloppE-Dip protocol overview above. The samples were shaken for 30 s and allowed to sit in the water/sediment slurry for either 0, 5, 10, and 20 min, ensuring that the FloppE-Dip membrane was always submerged (Table 2). After each settling time was up, the membranes were retrieved and stored according to the protocol outlined previously until extraction [35]. Duplicate negative control samples used 6 ounces of fresh bottled water without sediment or frog slurry. The membranes were shaken and allowed to sit for 20 min before membrane retrieval and storage.

**Table 2:** The sampling protocol for the FloppE-Dip samples (FD 1-4 and FD Blank) as well as the filtration samples (F1 and F Blank) showing the shake time and settling or contact times. Four replicates of each sediment sample were collected and two replicates of each blank.

Test name	Shake time (s)	Contact time (hh:mm)	Replicate (n)
FD 1	30	00:00	4
FD 2	30	00:50	4
FD 3	30	00:10	4
FD 4	30	00:20	4
FD Blank	30	00:20	2
		**Settling time** **(hh:mm)**	
F 1	30	24:00	4
F Blank	30	24:00	2

For the conventional filtration method, a 1 L polyethylene bottle was used instead of a baggie. Spiked sediment samples were suspended in 500 mL of dH_2_O, shaken for 30 s, and allowed to settle at 4°C for 24 h as described previously [13]. The samples were then filtered through a 0.45 m mixed nitrocellulose filter (ThermoFisher Scientific, Ottawa, ON, Canada) for up to 30 min. Duplicate 500 mL filtration blanks were prepared by filtering bottled water in the same manner. The filters were stored with silica beads at −20°C in a manual defrost freezer until DNA extraction [35].

### Field Application: Intertidal Sediment Samples

**Fig 2:**
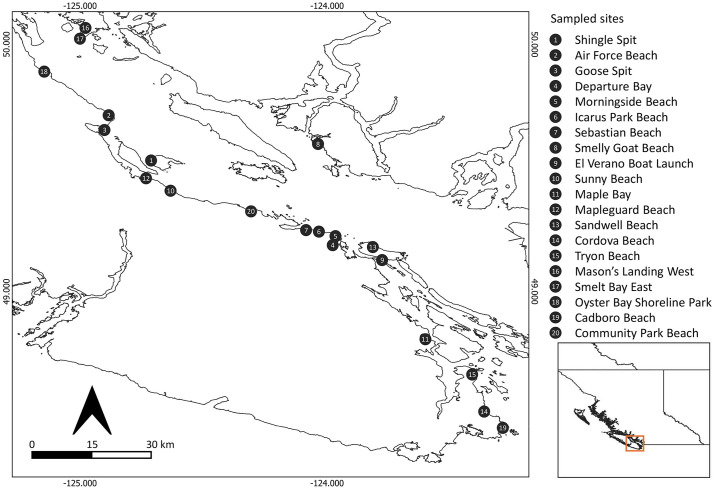
A field site map showing the locations where sediment samples for conventional filtration and FloppE-Dip samples were collected from beaches along the Salish Sea, British Columbia, for eDNA analysis. Latitude and longitude are indicated on the x and y axes.\ The map lines delineate study areas and do not necessarily depict accepted national boundaries.

Twenty different beaches potentially used by Pacific sand lance (*Ammodytes personatus)* in the Salish Sea (Fig 2) were sampled in December 2023 during the peak intertidal spawning window [36]. The known historical occurrence of Pacific sand lance as well as visual sightings of Pacific sand lance individuals or eggs at the time of sampling was obtained and tabulated for each site. At each site, a FloppE-Dip and a sediment sample for conventional eDNA filtration were collected in a pairwise manner and handled as described above but without the addition of frog slurry. Shake time was 30 s and settle time was 10 min. For sediment samples, 150 mL of sediment was collected into a plastic resealable bag. FloppE-Dip and sediment samples were transported to the lab with ice packs to keep them cool, where they were filtered as described above. All membranes and filters were stored with silica beads at −20°C in a manual defrost freezer until DNA extraction.

### DNA isolation

All samples were randomized and assigned sample processing numbers to reduce processing bias. DNA was isolated from one quarter of a FloppE-Dip membrane or conventional filter using the DNeasy Blood and Tissue kit (Qiagen Inc., Mississauga, ON, Canada; Cat# 69506) using previously described methods [11]. DNA sample eluate from the spin column (150 μL in Buffer AE) was stored at −20°C in a manual defrost freezer until qPCR analysis.

### qPCR analysis

The present study utilized three designed and validated assays as described previously [9,11,13,38–40]: IntegritE-DNA® assay (aka ePlant5) (detects chloroplast DNA), eLICA5 (detects *R. catesbeiana)*, and eAMPE5 (detects *A. personatus*). The reporting and performance characteristics of all three assays meet or exceed the criteria outlined in the Canadian eDNA national standards [1,37] and the technical bulletins of all three assays are in S2 file as S1 Fig, S2 Fig, and S3 Fig.

In applying the IntegritE-DNA® quality control step, a consistent threshold of C_q_ = 27 for filter and C_q_ = 30 for FloppE-Dip was set below which a sample replicate would be deemed as a “pass”. The three C_q_ difference accounts for the empirically determined ~10-fold difference in sensitivity between the two methods. These threshold values were established through the analysis of positive control signals and background noise. The IntegritE-DNA® assay detects chloroplast DNA which is ubiquitous in the environment and is present at high concentrations in eDNA samples [11,39]. All eDNA samples are run on this assay, thus acting as a naturally occurring and reliable endogenous positive control. If chloroplast DNA is not detected to an appropriate level, then the sample is deemed as poor quality due to inhibition, degradation, or other issues compromising the template DNA. If a sample fails the IntegritE-DNA® test, it will undergo an inhibitor removal step and be retested with the IntegritE-DNA® assay. If a sample still did not pass this quality control step, its data is considered unreliable as false negatives or an underestimation of eDNA copy number would be expected [13,39].

Two *μ*L of isolated eDNA sample was added in each 15 *μ*L qPCR reaction per well in a 96-well plate on a BioRad CFX96 thermocycler utilizing a three-step cycling regime: initial denaturation at 95°C for 9 min followed by 50 cycles of 15 s denaturation at 95°C, 30 s annealing at 64°C, and 30 s elongation at 72°C. The reactions contained 3 mM Mg^2+^, 250 *μ*M of each deoxynucleotide 5’ -triphosphate (dNTP), 1-unit Immolase Taq DNA polymerase (Froggabio), 700 nM of each primer and 10 nM hydrolysis probe (Integrated DNA Technologies, Coralville, Iowa, USA), and DNase-RNase-free ultrapure distilled water (UPdH_2_O) (Invitrogen, Massachusetts, USA). We prepared four technical replicates per sample for the IntegritE-DNA^TM^ assay and eight technical replicates per sample for the target assays. Each qPCR plate was run with both positive and negative controls: eight technical replicates of the no-template control (2 *μ*L UPdH_2_O) and two technical replicates of 20 copies/reaction of the appropriate gBlocks® - synthetic dsDNA identical to the sequence of target amplicon (Integrated DNA Technologies, Coralville, Iowa, USA).

### DNA copy number quantification

For samples with 100% detection (8/8 hits for the qPCR technical replicates), the estimated copy number per liter (or per sample) and standard error per liter (or per sample) was obtained using previously generated standard curves for each assay (IntegritE-DNA®, eLICA5, and eAMPE5) (S1 Fig, S2 Fig, S3 Fig in S2 File). For samples with <95% detection, the estimated copy number per liter (or per sample) and the standard error per liter (or per sample) was determined through the eDNA low copy number quantification (eLowQuant) R code, which utilizes a modified Binomial-Poisson statistical model [41] for the eLICA5 and eAMPE5 assays (S2 Fig and S3 Fig in S2 File).

### Statistical analyses

All statistical analyses were conducted using the R statistical package version 4.2.2 with an alpha level of 0.05 [42]. The Shapiro-Wilk normality test assessed whether the data followed a normal distribution. As the data were not normally distributed, the paired Wilcoxon test was used to determine statistical significance. Standard errors of the mean were calculated and reported where appropriate.

### Ethics statement

The tadpole was treated in accordance with the Canadian Council on Animal Care and the University of Victoria Animal Care Committee guidelines under protocol #2019−025.

## Results

### Spiked sediment experiment

The estimated copy numbers ± SE (copies/L) were determined for each FloppE-Dip sample, and an average was taken across all four replicates for each contact time point (Table 3, S1 Table in S2 File). The FloppE-Dip samples ran on the IntegritE-DNA® assay obtained comparable copy number values for all four contact time points with a mean ± SE of 141,880 ± 6,274 copies/L (n = 16). The average estimated copy number for the filter samples filtered for 30 min was ~ 5-fold higher at 673,536 ± 19,252 (n = 4) copies/L (Table 3). The copy numbers obtained for the FloppE-Dip samples run on the eLICA5 assay were also quite comparable for all four time points with a mean ± SE of 5,822 ± 1,130 copies/L (n = 16). The conventional filter sample produced an average copy number one order of magnitude lower than that of the FloppE-Dip samples at 372 ± 75 copies/L (n = 4) (Table 3). While all FloppE-Dip time points yielded comparable results, the 10 min time point was selected for the subsequent field sample testing due to the best combination of copy numbers returned with the lowest variation in the four replicates.

**Table 3:** The average estimated copy number (copies/L) and corresponding standard error (SE) for sediment samples spiked in American bullfrog tissue slurry collected via the FloppE-Dip method (FD) or conventional filtration (F). The FD membranes had a contact time with the spiked sediment in water for either 0, 5, 10, or 20 min and the F sample was filtered for 30 min. All samples were collected in quadruplicate.

Sample type	Number of replicates (n)	Contact or settling time (min)	IntegritE-DNA^®^ Average±SE (Copies/L)	eLICA5 Average±SE (Copies/L)
**FD**	4	0	152,870 ± 8,381	5,308 ± 2,137
	4	5	125,981 ± 6,010	7,243 ± 2,907
	4	10	145,072 ± 16,506	5,509 ± 2,693
	4	20	143,598 ± 16,719	5,228 ± 2,090
**F**	4	24 h	673,536 ± 19,252	372 ± 75

### Field Application: Intertidal sediment samples

#### Intertidal samples: IntegritE-DNA® assay.

**Fig 3:**
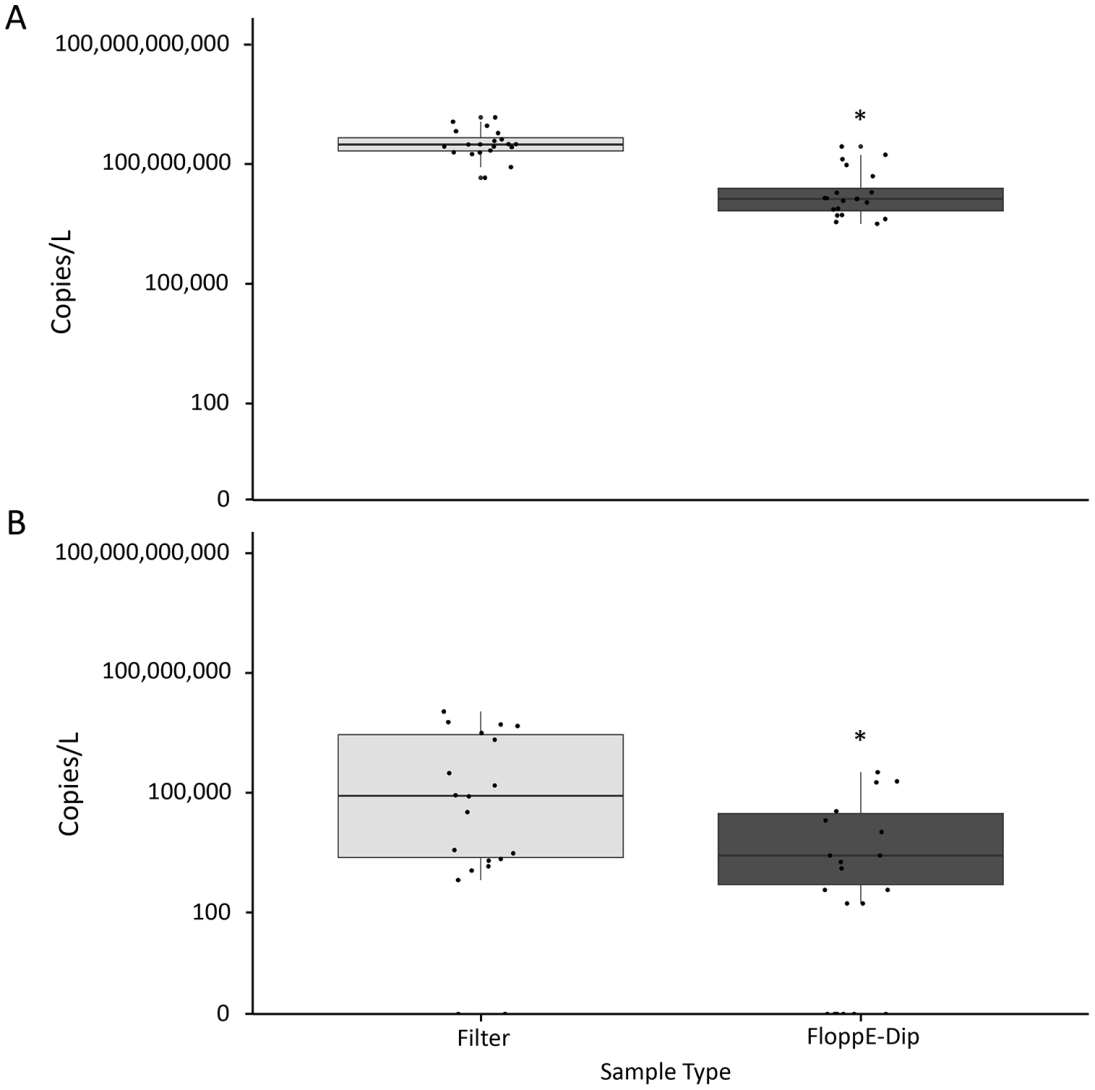
A box plot of the two data sets obtained from the filter samples and FloppE-Dip samples collected from beaches along the Salish Sea, British Columbia. The copy number (copies/L) distribution and variability within each sample type and between the sample types is shown. A) IntegritE-DNA® and B) eAMPE5 qPCR assays. Significant differences between pairwise comparisons using the Wilcoxon test are indicated by “*”.

Twenty field sediment samples were examined using conventional filtration and FloppE-Dip sampling methods. During the sampling periods, no rain events occurred. Two samples (Tryon Beach and Manson’s Landing West) required inhibitor clean up before passing the IntegritE-DNA® test (Table 4). All four technical replicates of all the samples (conventional filter and FloppE-Dip alike) produced C_q_ values below the positive detection thresholds (C_q_ = 27 for filter samples, C_q_ = 30 for FloppE-Dip samples) with strong signals (Table 4). The mean estimated average copy number ± SE (copies/L) for all 20 field samples run on the IntegritE-DNA® assay was 327,949,896 ± 62,747,818 copies/L for filter samples and 33,409,831 ± 12,258,540 copies/L for FloppE-Dip samples. Thus, the mean copy number for the FloppE-Dip samples was about 10-fold lower than that of conventionally filtered samples when testing with IntegritE-DNA® (Fig 3A). Additionally, the sample types showed significant differences in copy number distribution according to the paired Wilcoxon test (V = 208, p-value = 5.72e-06) (Fig 3A).

**Table 4:** The average estimated copy number and corresponding standard errors (copies/L) for each intertidal sand sample ran on the IntegritE-DNA® and the eAMPE5 qPCR assays in the Field Application experiment.

					IntegritE-DNA®				eAMPE5					
					**Filter**		**FloppE-Dip**		**Filter**		**FloppE-Dip**		**Visual Observation**	
**Sample ID**	**Site**	**Latitude**	**Longitude**	**Collection Date**	**Freq (/4)**	**Average±SE** **(Copies/L)**	**Freq (/4)**	**Copies/L**	**Freq (/8)**	**Average±SE** **(Copies/L)**	**Freq (/8)**	**Averahe±SE** **(Copies/L)**	**Historical Occurrence of Pacific Sand Lance**	**Pacific Sand Lance Found at Time of Sampling**
1	Shingle Spit	49.536575	−124.711536	05-Dec-23	4	456,925,026 ± 3,031	4	8,279,448 ± 65	8	2,156,647 ± 185,630	8	10,436 ± 2,150	Yes	Yes
2	Air Force Beach	49.720379	−124.885764	08-Dec-23	4	201,584,422 ± 2,637	4	14,514,641 ± 451	8	311,408 ± 40,209	8	328,980 ± 29,749	Yes	Yes
3	Goose Spit	49.661998	−124.905694	08-Dec-23	4	1,132,415,934 ± 7,902	4	4,007,197 ± 49	8	11,011,952 ± 1,572,167	8	183,014 ± 44,798	Yes	Yes
4	Departure Bay	49.205777	−123.969564	07-Dec-23	4	240,563,855 ± 2,804	4	10,693,932 ± 90	6	1,995 ± 961	2	367 ± 283	Yes	Yes
5	Morningside Beach	49.230852	−123.965188	07-Dec-23	4	695,255,666 ± 4,569	4	3,181,583 ± 27	8	153,064 ± 22,335	6	1,833 ± 883	Yes	Yes
6	Icarus Park Beach	49.2477238	−124.032868	07-Dec-23	4	509,978,459 ± 7,954	4	130,033,416 ± 2,335	8	86,671 ± 12,872	8	20,282 ± 2,691	Yes	Yes
7	Sebastian Beach	49.255718	−124.081419	07-Dec-23	4	239,401,235 ± 2,067	4	9,141,556 ± 128	8	81,572 ± 10,394	7	2,667 ± 1,350	Yes	Yes
8	Smelly Goat Beach	49.60525	−124.04205	08-Dec-23	4	150,650,273 ± 1,541	4	5,522,077 ± 54	8	5,204,943 ± 1,276,356	2	367 ± 283	Yes	Yes
9	El Verano Boat Launch	49.1344899	−123.778065	08-Dec-23	4	63,787,711 ± 1,421	4	5,824,947 ± 26	6	2,188 ± 1,054	1	167 ± 183	Yes	Yes
10	Sunny Beach	49.4074708	−124.633912	07-Dec-23	4	210,819,495 ± 1,121	4	10,598,513 ± 24	8	32,780 ± 4,384	5	1,267 ± 650	Suspected	Yes
11	Maple Bay	48.815741	−123.609537	07-Dec-23	4	149,089,766 ± 692	4	3,912,967 ± 27	0	0 ± 0	0	0 ± 0	Yes	No^a^
12	Mapleguard Beach	49.4657131	−124.735817	07-Dec-23	4	236,758,017 ± 1,451	4	14,891,688 ± 217	8	5,940,765 ± 2,406,799	8	194,904 ± 35,293	Yes	No^b^
13	Sandwell Beach	49.1865466	−123.819694	08-Dec-23	4	166,960,529 ± 4,467	4	210,873,816 ± 7,403	5	1,434 ± 736	0	0 ± 0	Yes	No
14	Cordova Beach	48.52485	−123.36551	19-Dec-23	4	290,674,164 ± 7,209	4	101,363,936 ± 1,595	4	1,125 ± 625	1	167 ± 183	No	No^c^
15	Tryon Beach^d^	48.67564	−123.408799	19-Dec-23	4	235,483,193 ± 2,968	4	2,674,941 ± 34	0	0 ± 0	0	0 ± 0	No	No^c^
16	Manson’s Landing West^d^	50.0663731	−124.981094	08-Dec-23	4	34,548,178 ± 642	4	10,128,602 ± 178	8	4,755,557 ± 3,688,067	7	2,667 ± 1,350	No	No
17	Smelt Bay East	50.03177	−124.99775	09-Dec-23	4	880,034,691 ± 20,817	4	72,230,045 ± 2,158	8	3,178,081 ± 808,284	8	34,456 ± 3,966	No	No
18	Oyster Bay Shoreline Park	49.896036	−125.146336	06-Dec-23	4	210,689,237 ± 4,466	4	10,195,135 ± 84	7	3,035 ± 1,537	0	0 ± 0	No	No
19	Cadboro Beach	48.459313	−123.289088	19-Dec-23	4	135,485,970 ± 4,435	4	37,718,629 ± 838	3	648 ± 414	0	0 ± 0	No	No
20	Community Park Beach	49.3256082	−124.309295	07-Dec-23	4	317,892,097 ± 2,844	4	2,409,555 ± 20	7	3,660 ± 1,853	0	0 ± 0	No	No^e^

**Sand for conventional filtration (Filter) and FloppE-Dip samples were collected in a pair-wise manner at 20 beaches on the Salish Sea, British Columbia. Visual observation recorded historically (as reported in S1 Table** in S2 File **Robinson et al. 2022 and from unpublished data from A. Vivani, Mount Arrowsmith Biosphere Region Research Institute) and during the sampling period are indicated for each site. Freq, Frequency; SE, standard error**

^a^Community sampling 4 days later found Pacific sand lance

^b^Community sampling 11 days later found Pacific sand lance

^c^Surf smelt were observed

^d^Required inhibitor clean up

^e^Community sampling a day later found Pacific sand lance

#### Intertidal samples: eAMPE5 assay.

Twelve of the 20 sites sampled along the Salish Sea coast had known historical occurrence of Pacific sand lance, one site was deemed as “suspected,” and the remaining seven sites had no known historical occurrence (based upon visual observation) (Table 4). At the time of sampling, Pacific sand lance eggs or adults were observed in 9/12 historically known sites as well as at the suspected site. No visual observations of sand lance were made at the other sites (Table 4).

Overall, the mean copy number (copies/L) for the eDNA samples run on the eAMPE5 assay was 1,646,376 ± 662,806 copies/L for filter samples and 39,079 ± 19,927 copies/L for FloppE-Dip samples, a ~ 40-fold difference in detection. Like the IntegritE-DNA® results, the filter samples yielded a higher mean estimated copy number than the FloppE-Dip samples (Fig 3B). A significant statistical difference was observed for the sample types by way of the paired Wilcoxon test (V = 163, p-value = 7.98e-04) (Fig 3B).

Both sample preparation methods detected Pacific sand lance DNA at 14 of the 20 sites sampled (Table 4). This includes all ten historically positive sites and one suspected site where Pacific sand lance was observed. Additionally, both methods also detected target DNA in four sites where Pacific sand lance were not observed at the time of sampling (Table 4). The conventional filtration method yielded a positive detection, but the FloppE-Dip did not at four sites (Sandwell Beach, Oyster Bay Shoreline Park, Cadboro Beach, and Community Park Beach). In those cases, the conventional filtration method resulted in fewer than 100% hits and 3,700 copies/L (Table 4). At one of those sites (Community Park Beach), community sampling a day later found sand lance adults (Table 4).

In two cases, Tryon Beach and Maple Bay, neither method detected sand lance DNA. Interestingly, surf smelt were observed at Tryon Beach, but no sand lance. At Maple Bay, no eggs or fish were found, but community sampling four days later visually observed sand lance at this site. These two observations reinforce the specificity of the eAMPE5 assay as well as accentuates the importance of temporal spacing of sampling events.

## Discussion

**Fig 4:**
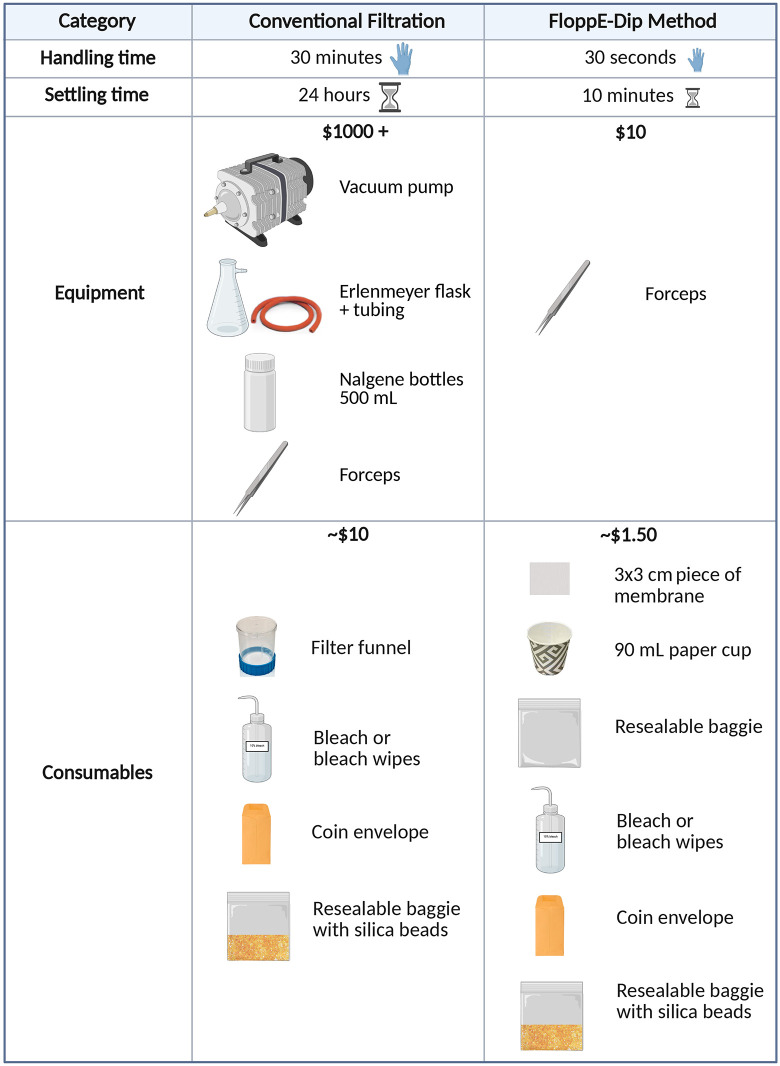
Comparison of time and monetary costs of conventional filtration versus the FloppE-Dip method. The basic equipment and consumables needed for each method are shown and the overall cost estimates in USD are indicated in bold.

Herein, we demonstrate that the FloppE-Dip method can support the detection of eDNA in sediment samples. Overall, the FloppE-Dip method yielded 5–10 times lower copies/L estimates than sediment samples taken through a conventional filtration method [12] but both were far more reliable than visual observation. Thus, FloppE-Dip can be used effectively with the caveat that for very low copy numbers (fewer than 100% hits and 3,700 copies/L for conventional filtration), it is possible to obtain false negative results as was seen in four of the sampling sites. However, increasing the number of sample replicates and/or the number of technical replicates can increase the statistical power and sensitivity of the FloppE-Dip method which could narrow the detection gap between the two methods [41]. The simplicity of the FloppE-Dip method for sample collection, reduced handling time (140X faster), and considerably reduced outlay in equipment costs (~100X cheaper) and consumables (~7X cheaper) make this a desirable approach (Fig 4).

As the present study was limited to sandy sediment, further evaluation of this method on diverse sediment types (e.g., muddy or rocky substrates, clay, or mixed matrices from wetlands, forests, and other marine or terrestrial environments) is needed. In another study, the FloppE-Dip method was able to detect eDNA of crab species from marine sediment collected around crab traps more effectively than observing crabs using conventional trapping methods, but not as effectively as metaprobe passive samplers deployed for 24 h or conventional filtration using water collected around the crab traps [43].

Nevertheless, the FloppE-Dip method is a viable option in situations for eDNA sediment or soil sampling with anticipated sample heterogeneity, where there is limited access to freezer space, and sampling budgets and resources are constrained. While we used cationic nylon membranes in the present study that facilitate eDNA capture through electrostatic interactions to the negatively charged DNA phosphate backbone, it is possible that uncharged nylon membranes could also be used, but this remains to be tested. The high mechanical strength of the nylon membrane enabled it to withstand handling and shaking.

## Conclusions

The present study demonstrates that the FloppE-Dip method can be used for effective eDNA detection. The IntegritE-DNA® quality control step shows that like conventional filters, FloppE-Dip samples can produce informative, high-quality results, albeit producing 5–10-fold lower copy number estimates compared to conventional filtration methods under controlled conditions. Despite this, the FloppE-Dip method provides substantial value in terms of accessibility and cost-effectiveness. Its simplicity makes it an ideal complementary tool for community-based monitoring and citizen science initiatives, enabling broader accessibility in eDNA sampling, rather than a replacement for conventional filtration, with future validations recommended across different membrane and sediment types. Additionally, the present study shows that it can be an attractive alternative to conventional methods specifically in contexts where the goal is to increase breadth and frequency of sampling while minimizing field time as well as for general detection purposes. We hope to empower citizen scientists and improve access to cutting edge biomonitoring techniques by strengthening low-cost, simple, and easily deployed approaches for eDNA sampling through the FloppE-Dip method.

## Supporting information

S1 File Step-by-step protocol, also available on protocols.io. (PDF)

S2 File Contains S1-S3 Figures and S1 Table. S1 Figure. Technical bulletin for performance characteristics of the IntegritE-DNA® eDNA assay. S2 Figure. Technical bulletin for performance characteristics of the eLICA5 eDNA assay. S3 Figure. Technical bulletin for performance characteristics of the eAMPE5 eDNA assay. S1 Table. Sample IDs, estimated copy numbers (copies/L), and standard errors (SE/L) obtained for American bullfrog spiked sediment samples ran on the IntegritE-DNA® and eLICA5 qPCR assays. Four FloppE-Dip (FD) samples and one filter (F) sample (four replicates of each) were collected, spiked with bullfrog standard slurry, and suspended in bottled water. The FD samples were exposed to the water/sediment and slurry mixture for either 0, 5, 10, or 20 min prior to DNA extraction. The F sample was settled in the mixture at 4°C for 24 h followed by 30 min of filtration. FD and F blanks contained only bottled water (no sediment or frog slurry).
(PDF)

S3 File Raw data for the manuscript. (XLSX)
