# Supplementary material for: Accessible and cost-effective deployment of environmental DNA (eDNA) samplers for sediment conducive to supporting community-based surveys
Source: PLoS One. 2026 Feb 24;21(2):e0342851. doi: 10.1371/journal.pone.0342851 (PMC12931753; doi:10.1371/journal.pone.0342851)
Supplement: S1 File — (PDF) [file pone.0342851.s001.pdf]

FloppE-Dip Method for Environmental DNA Sampling

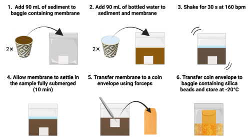

RESERVED DOI:
10.17504/protocols.io.261ge8q5og47/v1

Anna Dema[1], Ellika M. Crichton[1], Neha Acharya-Patel[1], Lauren C. Bergman[1], Michael J. Allison[1], Matthew T. Bonderud[1], Jacob Imbery[1], Clifford L.K. Robinson[2], Jacqueline R. Huard[3], Caren Helbing[1]

[1]University of Victoria; [2]Fisheries and Oceans Canada; [3]Comox Valley Project Watershed Society

Jacob Imbery
University of Victoria

Protocol Info: Anna Dema, Ellika M. Crichton, Neha Acharya-Patel, Lauren C. Bergman, Michael J. Allison, Matthew T. Bonderud, Jacob Imbery, Clifford L.K. Robinson, Jacqueline R. Huard, Caren Helbing . FloppE-Dip Method for Environmental DNA Sampling. **protocols.io** **https://protocols.io/view/floppe-dip-method-for-environmental-dna-sampling-g3uibynuf**

Created: June 23, 2025

Last Modified: November 24, 2025

Protocol Integer ID: 220778

Keywords: citizen science; community sampling; filtration; membrane-based eDNA detection; passive sampling; sandy sediment

Funders Acknowledgements:
Genome Canada
Grant ID: #312ITD
Genome British Columbia
Grant ID: #312ITD
Genome Quebec
Grant ID: #312ITD
Natural Sciences and Engineering Research Council of Canada (NSERC)
Grant ID: CGS-D graduate fellowships

Abstract

Rapid, on-site sampling of environmental DNA (eDNA) from sand, sediment, or water samples.

Image Attribution

The image was made by A. Dema.

Guidelines

As with any eDNA sampling protocol, best practices to reduce contamination should be followed and users are strongly encouraged to refer to the minimum reporting requirements of eDNA surveys for information regarding survey design and reporting [1] and that the eDNA assay for intended use meets the minimum performance criteria [2].

Materials

To prepare the FloppE-Dip membranes, you will need:

- Bleach wipes (e.g., Clorox) or 10% (v/v) bleach solution
- 70% (v/v) ethanol solution
- Positively charged membrane, **Cytiva LifeSciences Cat#60207 for roll**; **ThermoFisher Cat#77016 for 8×12cm sheet**
- Paper cutter or scissors
- Heavy-duty resealable plastic baggie

For each sample, you will need:

- 1 medium heavy-duty resealable plastic baggie containing one FloppE-Dip membrane
- 1 clean, unused 90 mL paper cup
- 1 small manila coin envelope
- 1 small heavy-duty resealable plastic baggie each containing ~1 tbsp of orange colour-indicating silica beads
- 1 - 3 X 3 cm piece of FloppE-Dip membrane

You will also need:

- Disposable nitrile or latex gloves
- 2 non-serrated medium tipped forceps
- Bottled or distilled water (180 mL per sample + additional for rinsing)
- Bleach wipes (e.g., Clorox) or 10% (v/v) bleach solution
- Timer
- Black, alcohol-resistant marker
- Cooler with ice packs for storage in the field after sampling
- 1 medium sized garbage bag for waste disposal

Troubleshooting

Safety warnings

When cutting the membrane, take care with the sharp blades.

Before start

Estimated time: 30 min per sampling event

Important notes and considerations:

To minimize the risk of contamination between samples:

- Wear a fresh pair nitrile or latex gloves to protect samples
- Do not reuse disposable sampling supplies for multiple samples (use paper cups and resealable baggies only once)
- Forceps should be disinfected with bleach wipes (e.g., Clorox wipes) or 10% (v/v) bleach solution, then thoroughly rinsed with distilled or bottled water before and in between sampling events.
- All sampling supplies should be disposed of properly after sampling.

Prepare your playlist (under Sampling method – Step 2) prior to heading out to the field!

Preparation of FloppE-Dip Kits

1 Don a fresh pair of nitrile or latex gloves.

2 Wipe work area with bleach wipes (e.g., Clorox) or 10% (v/v) bleach solution followed by 70% ethanol to remove bleach residue.

3 Sterilize blade (e.g., scissors, paper cutter) using bleach wipes (e.g., Clorox) or 10% (v/v) bleach solution, thoroughly remove bleach residue with distilled or bottled water either by rinsing or using a clean, wet, paper towel/Kimwipe. Allow to air dry thoroughly. Take care with the sharp blade.

4 Using the sterilized blade, cut 3×3 cm pieces of the positively charged membrane. The white membrane is sandwiched between protective pieces of paper. Ensure that the membrane is protected while cutting. Place pre-cut pieces into clean, individual re-sealable plastic baggies with the protective sheet surrounding the membrane still intact. Store at room temperature and away from light and noxious chemicals. Prepare a few extra than your planned number of samples.

5 Prepare 90 mL paper cups (one per sample) plus a few extra.

6 Prepare bleach wipes (e.g., Clorox) or 10% (v/v) bleach solution for decontamination between samples.

7 Prepare distilled or bottled water ensuring you enough for each sample (~180 mL per sand or sediment sample) and for rinsing the forceps between samples.

8 Prepare two clean non-serrated medium tipped forceps.

Preparation prior to field sampling

9 Don a fresh pair of nitrile or latex gloves.

10 Pre-label coin envelopes and resealable plastic baggies containing a single FloppE-Dip membrane with their respective sample IDs and the date of sampling using the black, alcohol-resistant marker. Double-check correct labelling.

11 Add ~1 tbsp silica beads to the appropriate number of new small resealable baggies. Make a few

extra.

Sampling

12 Transfer sample to plastic baggie containing FloppE-Dip:

12.1 For sand or sediment:

1. Using a clean 90 mL paper cup, measure 2 full, level scoops (180 mL) of sand and add to the resealable plastic baggie that contains a FloppE-Dip membrane.
2. Using the same paper cup, pour 2 full scoops (180) of distilled or bottled water into the same baggie. Remove the air and firmly seal the baggie.

12.2 For water:

1. Using a clean 90 mL paper cup, measure 4 full scoops (360 mL) of water and add to the resealable plastic baggie that contains a FloppE-Dip. Remove the air and firmly seal the baggie.

13 Shake the bag for 30 seconds at 160 beats per minute (BPM) (60-80 shakes). To help keep the beat, we have a curated a playlist of songs at ~160 BPM to shake along to – the playlist can be located here on Spotify: **FloppE-Dip Playlist**. To verify shaking rate, use a metronome or smartphone metronome app.

14 Allow sample to settle for 10 minutes. Use a timer to keep track. Ensure the membrane is always submerged in the sediment/water.

15 While waiting, sterilize the 2 forceps with your chosen bleach disinfectant, then rinse thoroughly with distilled or bottled water. Allow to air dry.

16 After the 10 minutes has passed, use the forceps to retrieve the membrane from the baggie and place it inside a pre-labelled coin envelope.

17 Put the coin envelope into a small re-sealable baggie containing ~1 tbsp of silica gel beads. Remove excess air and seal.

18 Place samples in a cooler with ice immediately and keep samples in the cooler during transfer from field to the lab.

19 Properly dispose of waste in between each sample and at the end of sampling.

20 For long term storage, store samples at -20°C in a manual defrost freezer until eDNA extraction.

Protocol references

1. Gagné N, Bernatchez L, Bright D, Côté G, Coulson M, Gurney K, et al. Environmental DNA (eDNA) reporting requirements and terminology. National Standard of Canada, CSA W214:21. National Standard of Canada. Ottawa: Canadian Standards Association; 2021. p. 31.
2. Abbott C, Bright D, Bryant H, Côté G, Crookes S, Gurney K, et al. Performance criteria for the analyses of environmental DNA by targeted quantitative polymerase chain reaction. National Standard of Canada, CSA W219:23. Ottawa: Canadian Standards Association; 2023. p. 29.
