# Supplementary material for: Accessible and cost-effective deployment of environmental DNA (eDNA) samplers for sediment conducive to supporting community-based surveys
Source: PLoS One. 2026 Feb 24;21(2):e0342851. doi: 10.1371/journal.pone.0342851 (PMC12931753; doi:10.1371/journal.pone.0342851)
Supplement: S2 File — S1 Figure. Technical bulletin for performance characteristics of the IntegritE-DNA® eDNA assay. S2 Figure. Technical bulletin for performance characteristics of the eLICA5 eDNA assay. S3 Figure. Technical bulletin for performance characteristics of the eAMPE5 eDNA assay. S1 Table. Sample IDs, estimated copy numbers (copies/L), and standard errors (SE/L) obtained for American bullfrog spiked sediment samples ran on the IntegritE-DNA® and eLICA5 qPCR assays. Four FloppE-Dip (FD) samples and one filter (F) sample (four replicates of each) were collected, spiked with bullfrog standard slurry, and suspended in bottled water. The FD samples were exposed to the water/sediment and slurry mixture for either 0, 5, 10, or 20 min prior to DNA extraction. The F sample was settled in the mixture at 4°C for 24 h followed by 30 min of filtration. FD and F blanks contained only bottled water (no sediment or frog slurry). (PDF) [file pone.0342851.s002.pdf]

Supplementary Information

Accessible and cost-effective deployment of environmental DNA (eDNA) samplers for sediment conducive to supporting community-based surveys

Anna H. Dema[1], Ellika M. Crichton[1], Neha Acharya-Patel[1], Lauren C. Bergman[1], Michael J. Allison[1], Matthew T. Bonderud[1], Jacob J. Imbery[1], Clifford L.K. Robinson[2], Jacqueline R. Huard[3], and Caren C. Helbing[1]*

[1]Department of Biochemistry and Microbiology, University of Victoria, 3800 Finnerty Road, Victoria, British Columbia V8P 5C2, Canada
[2]Pacific Biological Station, Fisheries and Oceans Canada, Nanaimo, British Columbia V9T 6N7, Canada
[3]Comox Valley Project Watershed Society, 2356 Rosewall Crescent, Courtenay, British Columbia V9N 8R9, Canada

*Corresponding Author:
Email: chelbing@uvic.ca

**Table of Contents:**	
**S1 Figure**	Technical bulletin for performance characteristics of the IntegritE-DNA® eDNA assay
**S2 Figure**	Technical bulletin for performance characteristics of the eLICA5 eDNA assay
**S3 Figure**	Technical bulletin for performance characteristics of the eAMPE5 eDNA assay
**S1 Table**	Sample IDs, estimated copy numbers (copies/L), and standard errors (SE/L) obtained for American bullfrog spiked sediment samples ran on the IntegritE-DNA® and eLICA5 qPCR assays. Four FloppE-Dip (FD) samples and one filter (F) sample (four replicates of each) were collected, spiked with bullfrog standard slurry, and suspended in bottled water. The FD samples were exposed to the water/sediment and slurry mixture for either 0, 5, 10, or 20 min prior to DNA extraction. The F sample was settled in the mixture at 4°C for 24 h followed by 30 min of filtration. FD and F blanks contained only bottled water (no sediment or frog slurry).

General eDNA Assay Information

Target Species: Chloroplasts (Plant/Algae) | eDNA qPCR Tool: ePlant5 | Gene Target: 23S rRNA

Species Code: IntegritE-DNA | eDNA qPCR Format: TaqMan | Published in: 1, 2, 3

eDNA Assay Sensitivity Test Summary using gBlocks™ Synthetic DNA

LOD N/A 95% CI N/A Copies | LOQ N/A 95% CI N/A Copies | LOB N/A hits/8

Binomial-Poisson model for 8 technical replicates

Determined using eLowQuant R code[4]. | When the LOQ < LOD, use the LOD for the LOQ. | Enzyme: Immolase

eDNA Assay Specificity Test Information

Each qPCR reaction in the specificity assay contained 10 picograms of voucher target gDNA (n=25 technical replicates)

Chloroplast DNA is used as an endogenous quality control for eDNA samples when testing for animal DNA.
A broad range of plants and algae from fresh water, marine water, sediments, air, and soil have been tested and pass.
Fresh, unopened bottled water typically does not have plant or algae DNA in it.
Typically the C_q cutoff is 27 for pass/fail for most applications.

"Fail" indicates poor quality sample with inhibition and/or degradation.
Refer fo references 1 and 2 for application examples.

The continuous standard curve is based upon n=8 technical replicates with very small standard error.
Based on a 2 µL DNA input in a total 15 µL reaction

Applied to reactions with 100% positive hits

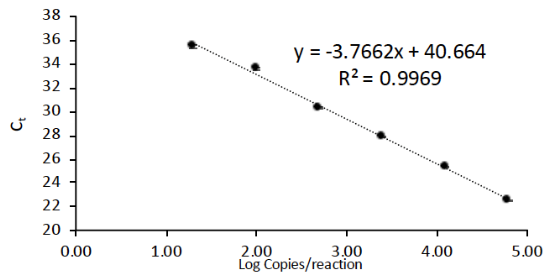

Efficiency 84%

Abbreviations

23S rRNA	23S ribosomal RNA	NTC	qPCR no template control
eDNA	Environmental DNA	qPCR	Quantitative real-time polymerase chain reaction
gDNA	Total genomic DNA extracted from voucher specimen	SE	Standard error

S1 Figure. Technical bulletin for performance characteristics of the IntegritE-DNA® eDNA assay.

General eDNA Assay Information

Target Species: North American Bullfrog *(Lithobates [Rana] catesbeiana)*
eDNA qPCR Tool: eLICA5
Gene Target: MT-ND5
Species Code: am-LICA
eDNA qPCR Format: TaqMan
Published in: 4

eDNA Assay Sensitivity Test Summary using gBlocks™ Synthetic DNA

LOD 0.7 95% CI 0.5-1.2 Copies LOQ 2.8 95% CI 2-4.5 Copies LOB 0 hits/8

$LOQ_{continuous}$ 20 Copies/Rxn

Binomial-Poisson model for 8 technical replicates determined using eLowQuant R code[4]. When the LOQ < LOD, use the LOD for the LOQ. Enzyme: Immolase

eDNA Assay Specificity Test Information

Each qPCR reaction in the specificity assay contained 10 picograms of voucher target gDNA (n=25 technical replicates)

Species	Common Name (*Species*)	Detection	# Voucher Specimens	Sample Sources/Locations
am-LICA	Bullfrog (*Lithobates (Rana) catesbeiana*)	Yes	5	British Columbia
am-ANBO	Western Toad (*Anaxyrus (Bufo) boreas*)	No	2	Northwest Territories
am-LICL	Green frog *(Lithobates [Rana] clamitans)*	No	1	British Columbia
am-LIPI	Northern leopard frog *(Lithobates [Rana] pipiens)*	No	2	Alberta
am-LISY	Wood frog *(Lithobates [Rana] sylvaticus)*	No	1	British Columbia
am-PSRE	Pacific chorus frog *(Pseudacris [Hyla] regilla)*	No	1	British Columbia
am-PSMA	Boreal chorus frog *(Pseudacris maculata)*	No	1	Ontario
am-RAAU	Northern red-legged frog *(Rana aurora)*	No	1	British Columbia
am-RACA	Cascades frog *(Rana cascadae)*	No	1	British Columbia
am-RALU	Columbia spotted frog *(Rana luteiventris)*	No	1	British Columbia
am-RAPR	Oregon spotted frog *(Rana pretiosa)*	No	1	British Columbia
am-SPIN	Great basin spadefoot toad *(Spea [Scaphiopus] intermontana)*	No	1	British Columbia
am-XELA	African clawed frog *(Xenopus laevis)*	No	1	South Africa
am-AMGR	Northwestern salamander *(Ambystoma gracile)*	No	1	British Columbia
am-AMMA	Long toed salamander *(Ambystoma macrodactylum)*	No	1	British Columbia
am-AMMV	Eastern tiger salamander *(Ambystoma mavortium)*	No	1	British Columbia
am-ANBO	Western toad *(Anaxyrus [Bufo] boreas)*	No	1	British Columbia
am-ANAM	American toad *(Anaxyrus americanus)*	No	1	British Columbia
ma-CALUfa	Canine (*Canis lupus familiaris*)	No	1	British Columbia
ma-FECA	Cat (*Felis catus*)	No	1	British Columbia
ma-HOSA	Human (*Homo sapiens*)	No	1	Netherlands

eDNA Assay Sensitivity Test Details using gBlocks™ synthetic DNA

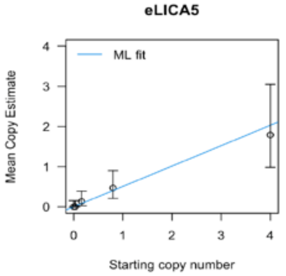

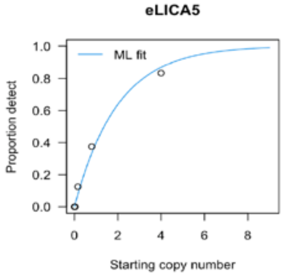

From 8 Technical Replicates

# Detects	# Copies	SE
0	0	0
1	0.26	0.27
2	0.57	0.42
3	0.93	0.57
4	1.37	0.75
5	1.93	0.98
6	2.73	1.32
7	4.10	2.01

Determined using eLowQuant R code[4].

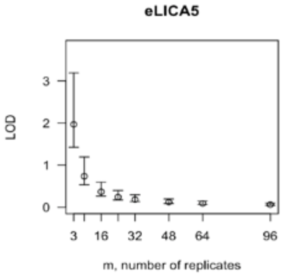

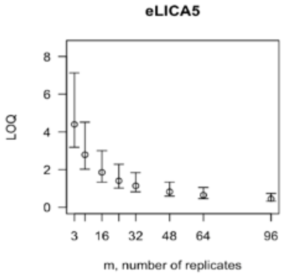

Applied to reactions with 100% positive hits

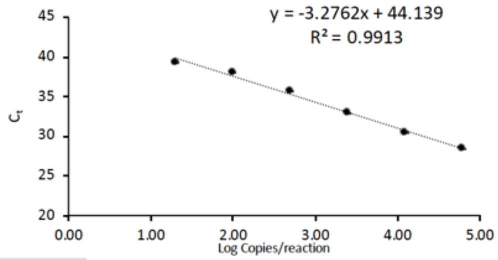

Binomial-Poisson model: No intercept
Determined using eLowQuant R code[4].
Based on a 2 µL DNA input in a total 15 µL reaction

Efficiency 102%

Field Sample Validation

Sample Type	Known Presence	# Samples	Detected	Location
Freshwater	Y	1	Y	Victoria, BC

Abbreviations

95% CI	95% Confidence interval	LOQ	Limit of quantification
eDNA	Environmental DNA	MT-ND5	Mitochondrial NADH dehydrogenase subunit 5 gene
gDNA	Total genomic DNA extracted from voucher specimen	NTC	qPCR no template control
LOB	Limit of blank	qPCR	Quantitative real-time polymerase chain reaction
LOD	Limit of detection	SE	Standard error

S2 Figure. Technical bulletin for performance characteristics of the eLICA5 eDNA assay.

General eDNA Assay Information

Target Species:	Pacific Sandlance (*Ammodytes personatus*)	eDNA qPCR Tool:	eAMPE5	Gene Target:	MT-RNR2 (16S)
Species Code:	te-AMPE	eDNA qPCR Format:	TaqMan	Published in:	3, 4

eDNA Assay Sensitivity Test Summary using gBlocks™ Synthetic DNA

LOD 0.3 95% CI 0.2-0.5 Copies/Rxn LOQ 1.1 95% CI 0.8-1.9 Copies/Rxn LOB 0 hits/8

$LOQ_{continuous}$ 4 Copies/Rxn

Binomial-Poisson model for 8 technical replicates determined using eLowQuant R code[4]. When the LOQ < LOD, use the LOD for the LOQ. Enzyme: Immolase

eDNA Assay Specificity Test Information

Each qPCR reaction in the specificity assay contained 10 picograms of voucher target gDNA (n=25 technical replicates)

Species	Common Name (*Species*)	Detection	# Voucher Specimens	Sample Sources/Locations
ma-HOSA	Human (*Homo sapiens*)	No	1	Netherlands
te-AMPE	Pacific Sand Lance (*Ammodytes personatus*)	Yes	5	British Columbia
te-HYPR	Surf Smelt (*Hypomesus pretiosus*)	No	2	British Columbia
te-ONGO	Pink Salmon (*Oncorhynchus gorbuscha*)	No	1	British Columbia
te-ONKE	Chum Salmon (*Oncorhynchus keta*)	No	1	British Columbia
te-ONKI	Coho Salmon (*Oncorhynchus kisutch*)	No	1	British Columbia
te-ONNE	Sockeye Salmon (*Oncorhynchus nerka*)	No	1	British Columbia
te-ONTS	Chinook Salmon (*Oncorhynchus tschawytscha*)	No	1	British Columbia
te-THPA	Eulachon (*Thaleichthys pacificus*)	No	1	British Columbia

eDNA Assay Sensitivity Test Details using gBlocks™ synthetic DNA

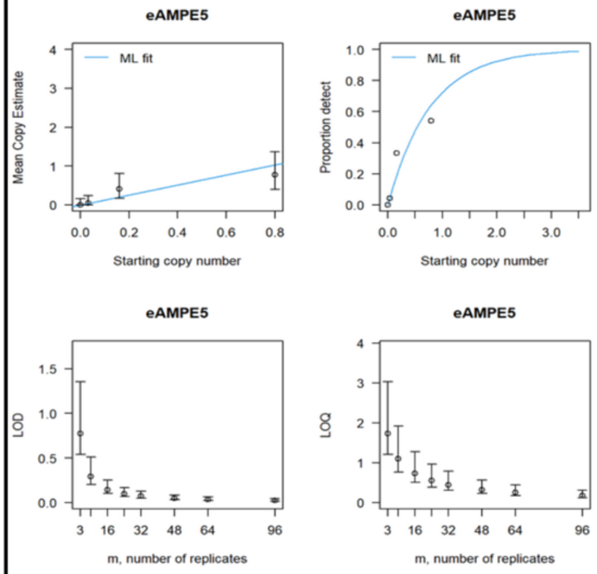

From 8 Technical Replicates

# Detects	# Copies	SE
0	0	0
1	0.1	0.11
2	0.22	0.17
3	0.36	0.23
4	0.54	0.3
5	0.76	0.39
6	1.1	0.53
7	1.6	0.81

Determined using eLowQuant R code[4].

Applied to reactions with 100% positive hits

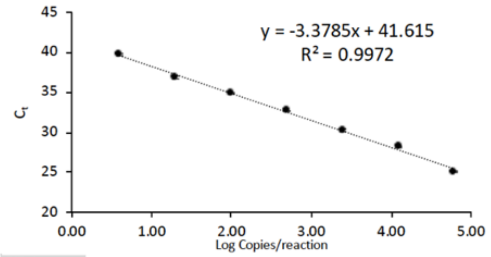

Efficiency 98%

Binomial-Poisson model: No intercept

Determined using eLowQuant R code[4].

Based on a 2 µL DNA input in a total 15 µL reaction

Field Sample Validation

Sample Type	Known Presence	# Samples	Detected	Location
Sand	Y	5	Y	British Columbia

Abbreviations

95% CI	95% Confidence interval	LOQ	Limit of quantification
eDNA	Environmental DNA	MT-RNR2 (16S)	Mitochondrial 16S ribosomal RNA
gDNA	Total genomic DNA extracted from voucher specimen	NTC	qPCR no template control
LOB	Limit of blank	qPCR	Quantitative real-time polymerase chain reaction
LOD	Limit of detection	SE	Standard error

S3 Figure. Technical bulletin for performance characteristics of the eAMPE5 eDNA assay.

S1 Table. Sample IDs, estimated copy numbers (copies/L), and standard errors (SE/L) obtained for American bullfrog spiked sediment samples ran on the IntegritE-DNA® and eLICA5 qPCR assays. Four FloppE-Dip (FD) samples and one filter (F) sample (four replicates of each) were collected, spiked with bullfrog standard slurry, and suspended in bottled water. The FD samples were exposed to the water/sediment and slurry mixture for either 0, 5, 10, or 20 min prior to DNA extraction. The F sample was settled in the mixture at 4°C for 24 h followed by 30 min of filtration. FD and F blanks contained only bottled water (no sediment or frog slurry).

Sample ID	Settling time (hh:mm)	IntegritE-DNA®			eLICA5	
		Pass or Fail	Copies/L	SE/L	Copies/L	SE/L
FD 1A	00:00	Pass	155,592	2,117	2,996	571
FD 1B		Pass	139,659	3,792	5,441	897
FD 1C		Pass	140,708	2,836	1,548	1,118
FD 1D		Pass	175,519	10,256	11,248	1,992
FD 2A	00:05	Pass	127,670	1,797	4,252	671
FD 2B		Pass	110,511	2,342	6,155	1,213
FD 2C		Pass	139,810	4,384	15,725	3,192
FD 2D		Pass	125,932	2,366	2,841	478
FD 3A	00:10	Pass	136,448	3,686	1,341	193
FD 3B		Pass	180,297	4,512	12,023	2,316
FD 3C		Pass	103,485	2,260	837	81
FD 3D		Pass	160,057	636	7,835	270
FD 4A	00:20	Pass	155,790	3,762	3,514	1,106
FD 4B		Pass	185,356	1,271	5,730	1,051
FD 4C		Pass	118,487	2,469	910	67
FD 4D		Pass	114,761	7,752	10,756	5,321
F 1A	24:00	Pass	631,180	18,753	491	253
F 1B		Pass	706,164	15,994	258	129
F 1C		Pass	706,164	15,994	228	376
F 1D		Pass	650,635	11,909	512	218
FD Blank-A	00:20	Fail	0	0	0	0
FD Blank-B		Fail	0	0	0	0
F Blank-A	24:00	Fail	0	0	0	0
F Blank-B		Fail	0	0	0	0

References

1. Hobbs J, Round JM, Allison MJ, Helbing CC. Expansion of the known distribution of the coastal tailed frog, *Ascaphus truei*, in British Columbia, Canada using robust eDNA detection methods. PLOS ONE. 2019;14(3):e0213849.
2. Hobbs J, Adams I, Round JM, Allison MJ, Goldberg CS, Helbing CC. Extension of the Rocky Mountain tailed frog range in British Columbia, Canada using eDNA methods. Environmental DNA. 2020;2:350-61.
3. Robinson CLK, Bergman LC, Allison MJ, Huard J, Sutherst J, Helbing CC. Application of environmental DNA as a tool for detecting intertidal habitat use by forage fish. Ecological Indicators. 2022;142:109306.
4. Langlois VS, Lopez MLD, Allison MJ, Imbery JJ, Couillard J, Acharya-Patel N, et al. Environmental DNA (eDNA) quantitative polymerase chain reaction-based assays for surveying 125 taxa of importance to North America. Environmental DNA. 2025;7:e70139.
